# Why is preconception health and care important?

**DOI:** 10.1080/03009734.2016.1211776

**Published:** 2016-08-02

**Authors:** Tanja Tydén

**Affiliations:** Professor and Chair, Caring Sciences Uppsala University, Uppsala, Sweden, Congress President

The third European Congress on Preconception Health and Care was held in Uppsala, Sweden in February 2016. The congress was hosted by the Medical Faculty at Uppsala University and the PrePreg Research Network. This informal network was established after the first European Congress on Preconception Health and Care in Brussels in 2010. The aim of the conference was to promote further international co-operation and knowledge sharing amongst researchers. By means of a generous grant from The Swedish Research Council to cover travel costs for invited key-note speakers it became possible for us to plan and realize the conference.

Preconception care is a set of interventions intended to identify and to modify biomedical, behavioural, and social risks in women of reproductive age. The goal of preconception care is to improve pregnancy outcomes and women’s health in general through prevention of disease and management of risk factors that affect pregnancy outcome and the health of future generations. In 2013, the World Health Organization (WHO) pointed out that preconception care is relevant for all women of reproductive age. In high-income countries women postpone childbearing until ages when their fecundity has decreased, whereas women in low-income countries would benefit from delaying pregnancy and spacing of subsequent pregnancies. Since the most critical period for organ development occurs before many women even know they are pregnant, the first contact with antenatal care is often too late for advice about health-promoting changes in lifestyle. Moreover, there is a growing body of evidence that women’s, and also men’s, health and lifestyle before conception can affect pregnancy outcomes. Globally, at least four out of ten women report that their pregnancies were unplanned, highlighting the need for population-wide approaches for evidence-based preconception care. Women with chronic diseases such as SLE and diabetes, hypertension, and obesity face unique reproductive planning challenges.

The specific aims of the congress were: (1) to review the status of preconception health and care in Europe and globally; (2) to present recent preconception health research results; (3) to exchange experiences with different methodologies used in clinical research, including issues relating to gender and ethics; (4) to exchange experiences of implementing and evaluating reproductive health care interventions, including the use of social media to promote preconception behaviour change; and (5) to develop new research collaborations.

I am happy that so many leading researchers within the field of preconception health and care participated and contributed to interactive discussions that brought the concept of preconception health and care forward. A wide range of aspects on preconception health and care were discussed during three days, such as:

- A global overview of preconception care, including information about the Zika infection
- Prevention of unwanted pregnancies
- Status of preconception health in the US and Europe
- Postponement of family formation, including new technologies and ethics
- Obesity and reproductive health
- Violence and abuse
- A worldwide estimate of the prevalence of congenital conditions
- Presentation of The Swedish Medical Birth Register
- Use of social media and the Internet to promote preconception care

You can find further information on the speakers’ presentations on the congress website (http://www.pubcare.uu.se/ecphc2016/) and in this special issue of *UJMS* where the key-note speakers were offered the opportunity to write an article on their research topics.

